# Attitude and prevalence of early sexual debut and associated risk sexual behavior among adolescents in Tanzania; Evidence from baseline data in a Randomized Controlled Trial

**DOI:** 10.1186/s12889-023-16623-6

**Published:** 2023-09-09

**Authors:** Walter C. Millanzi, Kalafunja M. Osaki, Stephen M. Kibusi

**Affiliations:** 1 Department of Nursing Management and Education, School of Nursing and Public Health, The University of Dodoma, Dodoma, Tanzania; 2 St. Augustine University of Tanzania, Dar Es Salaam, Tanzania; 3 The University of Dodoma, Dodoma, Tanzania

**Keywords:** Adolescent, Sexual behavior, Sexual ideology, Sexual debut, Tanzania

## Abstract

**Background:**

Unsafe sexual behaviours and associated sexual ideas among adolescents may contribute to adverse health consequences for sexual health in adulthood. The patterns of sexual ideology and sociodemographic factors profiles on adolescents' sexual behaviours have not been the subject of a definite consensus in research. The purpose of this study was to investigate the attitude and prevalence of early sexual debut and associated risk sexual behavior among adolescents in Tanzania as the evidence from baseline data in a Randomized Controlled Trial.

**Methods:**

The study included 647 randomly chosen in-school adolescents from Tanzania and used an analytical cross-section survey in a quantitative research approach. Sexual-risk Behaviour Beliefs and Self-esteem Scale from previous studies were the main data collection tool. According to the Statistical Analysis Software (SAS), computer software version 9.4 descriptive analysis established respondents' socio-demographic profiles, attitudes, prevalence, and determinants linked to teenagers' early sexual debut. The link between the variables was established via multivariate logistic regression at a 5% significance level and a 95% confidence interval.

**Results:**

The mean age was 15 ± 1.869 years while 57.5% of adolescents were females. 69.7% of adolescents were sexually active whereas 44.8% of them practised sexual behaviours willingly against 24.9% who practised coerced sexual behaviours. The majority (44.4%) and 16.2% of them initiated sexual behaviours during the early and middle adolescence stages respectively. Most adolescents had the ideology that sex was okay to them even before the age of 18 years. Their odds of practicing sexual behaviours were significantly high with the ideology that sex was okay to them even before 18 years of age (AOR = 1.293; *p* < 0.05; 95%CI: 0.689, 2.989), exposure to drug abuse (AOR = 1.210; *p* < 0.05; 95%CI: 0.803, 2.130), using media (AOR = 1.006; *p* < 0.05; 95%CI: 0.748, 2.667) and/or exposure to social groups [Jogging, Gym, health clubs, betting, Games] (AOR = 1.032; *p* < 0.05; 95%CI: 0.889, 2.044).

**Conclusion:**

Findings suggest that holding a positive attitude towards early sexual debut is a precursor to early sexual activity among adolescents. Unsafe sex, coercive sex, and other risky sexual behaviors are not uncommon among adolescents starting sex before the age of 18 years. Exposure to drug abuse, online sexual content, and/or social groups significantly influenced early sexual debut irrespective of other known factors. Age-appropriate school-based sexuality education programs should be promoted and implemented to address the most prevalent positive attitude towards early sexual debut and associated risk sexual behaviour among adolescents in Tanzania and other similar settings.

## Introduction

Adult sexual health effects may be closely linked with unsafe sexual activities associated with young people's sexual views, norms, and self-efficacy during adolescence stages [1]. Sexual health here means a state of physical, emotional, mental, and social well-being regarding sexuality. Additionally, it promotes a positive outlook on sexuality and sexual relationships as well as the potential of enjoying enjoyable and safe sexual experiences free from prejudice, compulsion, and violence [2]. Contrarily, unsafe sexual behaviour includes using contraceptives incorrectly or inconsistently, having several partners, having unprotected sex often, and/or using drugs or alcohol before or during sexual activity [3]. However, it has been determined that the presence of a robust youth generation globally is predicted by effective formation tactics that gather a healthy cohort of young people [1, 4]. When combined and connected with a coherent and positive self-identity, the adolescent's capacity to integrate his or her sexual feelings, needs, and desire which determine the sexual self is essential [5].

Even if cultural expectations and the sexual discourse typical of social situations appear to have a complex influence on the sexual self, it is crucial to reveal how young people define the sexual worlds that have developed in their lives [6]. As estimated by various scholars [7], one of the main factors influencing people's ability to lead healthy lives is safe sexual behaviours. However, teens (10 to 19 years) are the most susceptible to the negative effects of unprotected sex because, according to UNICEF, these behaviours are linked to two-thirds of adult premature deaths and one-third of the entire burden of diseases [8]. Drug use, poor parenting, peer pressure, sociocultural standards, mass media, and individual sexual attitudes are all occasionally associated with the behaviours [9, 10]. Unsafe sexual behaviours are a health determinant that is frequently connected to unintended pregnancies and the obstetric outcomes they are associated with, such as anemia, puerperal psychosis, eclampsia, fistula, abortion, and hemorrhage, to name a few, as well as new STIs/HIV infections [11, 12].

Teenagers on the streets, in schools, and those whose parents consent to early marriages face the constant threat of engaging in risky sexual behaviours that could expose them to sexual masculinity, gender-based sexual abuse during adolescence, unwanted teenage pregnancies, early marriages, and/or dropping out of school, all of which are frequently done without their consent [13, 14]. In low-and-middle-income nations Tanzania inclusive, for instance, early adolescent marriages are always forbidden and take place behind the consent of adolescent girls and at the families' discretion at the beginning of and just before menstruation [15]. The issue has traditionally been associated with teenagers' diminished capacity for negotiation and their informed, deliberate, and responsible decisions regarding sexual activities, including coercive and unsafe sexual actions [16, 17].

Findings from previous scholarly works [18, 19] also, demonstrate a decline in several nations in the rates of teenage pregnancies before the age of 15 years. However, the rate of early pregnancies before the age of 18 remains largely stable [20]. It is widely accepted that the global HIV incidence has decreased from 0.40 per 1000 populations without the disease in 2005 to 0.26 per 1000 populations without the disease in 2016 [21]. The situation is different in African regions, where the incidence rate of HIV has reached 1.24 per 1000 inhabitants who are not affected, and where an estimated 1 million people have died of illnesses associated with HIV [22]. An estimated 120,000 teenagers (10 to 19 years old) have died of AIDS-related illnesses. In addition, studies indicate that SSA has a high rate of new HIV infections (7.3% of males and 31.8% of females between the ages of 15 and 24) [23].

Tanzania, one of Sub-Saharan Africa's developing nations, has the 17th-highest adolescent fertility rate on the continent [24]. Teenage pregnancies have increased by 4%, and the adolescent fertility rate climbed from 116 to 132 between the Demographic Health Surveys (TDHS) conducted in 2010 and 2015/2016 [25]. According to the trend, one in four teenagers between the ages of 15 and 19 had started having children, likely as a result of the early commencement of risky sexual behaviours [2, 26]. Regional differences may be seen in Tanzania's childbearing rates, which range from lows of 5% in Zanzibar's Mjini Magharibi region and 6% in the Kilimanjaro region to highs of 45% in Katavi and 43% in Tabora. Approximately 39% of the country's teenage childbearing rate was in the Dodoma and Morogoro regions [27]. Additionally, research shows that adolescents in rural areas were noted to be more likely to start having children (32%) than their urban counterparts (19%) [9]. Despite these realities, young people especially girls are blamed by parents and other community members for engaging in early sexual activity with older men and boys their age purely for selfish financial reasons [1].

The reasons for adolescents' risky sexual behaviours in the nation seem to be unclear Wado et al*.,* [28]. By limiting conversations about sexual and reproductive health and influencing young people's engagement in risky sexual behaviours, for instance, parenting and family structure have been shown to affect young people's confidence and social competence. Additionally, it was discovered that the power of men influenced how girls negotiated their sexual interactions. Teenagers are prematurely exposing themselves to STIs and HIV as a result, as well as teenage pregnancies, school dropouts, early marriages, and related obstetric complications such as abortions, fistulas, post-partum hemorrhage (PPH), and early mother mortality [29, 30]. Teenagers are also exposed to health risks due to the dissolution of old social and economic institutions, growing urbanization, and greater population movement worldwide [31]. Teenagers are more likely to engage in risky sexual behaviours because of the prevalence of commercial sex that takes place online, illegal and potentially harmful drug usage, and sexual harassment (sexual onset, rape, violence, and mistreatment) [32].

However, experts and educators in the field of sexuality have identified sexual ideology as a determinant of self-esteem, which serves as a barrier to a range of risky sexual behaviours among teenagers [33]. It is important to note that whether someone exhibits self-rejection, self-contempt, and self-dissatisfaction or not depends on their level of self-esteem as a result of having good sexual ideas [4]. Here, the term "sexual ideas" refers to one's beliefs, perceptions, and/or feelings about oneself as a sexual being, which may be good or negative [34, 35]. Good sexual ideals may be associated with self-control, moral integrity, societal responsibility, and making thoughtful, responsible judgments about sexual relationships and sexual behaviour [10, 30]. There appear to be outstanding problems regarding the generalization that sexual ideology influences adolescents' sexual behaviour based on previously reported reports and conclusions.

Furthermore, it appears that there is not enough quality research that concentrates on teenagers in elementary/ordinary schools such as primary and secondary schools. Elementary/ordinary schools here mean fundamental education based on the education system of Tanzania, in which most early (10 – 13 years.) and middle aged (14 – 18 years.) adolescents are admitted. However, there is still no universally accepted theory explaining how sexual ideology and teenage sexual behaviour are related. The goal of the current study was to address and close this apparent gap in knowledge through the four specific objectives including; assessing sexual ideology, prevalence, onest, and factors associated with sexual behaviours among in-school adolescents in Tanzania. The authors of this study were interested in learning how adolescents perceive sexual behaviours/activities, what percentage of teenagers engage in sexual behaviours? When do teenagers first engage in sexual activity? And what circumstances motivate teenagers to engage in sexual behaviour/activity?

## Materials and methods

The rules and criteria for undergraduate and graduate programs at the institution that serve as a foundation for researching while adhering to ethical considerations to meet national and international research standards were used in the conduct of this study [36].

### Study design

The study employed an analytical cross-sectional to quantify in-school adolescents’ attitude and prevalence of early sexual debut and associated risk sexual behavior among adolescents in Tanzania as the evidence from baseline data in a Randomized Controlled Trial.

### Study Sample

This study involved 647 adolescents including those in early adolescence (10 to 12 years); middle adolescence (13 to 16 years) and late adolescence (17 to 19 years) ( [1, 4]. In-school adolescents have primarily opted for because schools were treated as ideal locations where with the support of teachers, authors would access, manage and assess them more easily at a single point in time. Furthermore, this study held that studying teenagers who are enrolled in school would be advantageous because it is more practicable to carry out educational interventions on a sizable sample of adolescents than on those who are not.

Nevertheless, the age range of 10 to 19 years adolescents was chosen because, according to the World Health Organization [37], it is a distinct stage of human growth and development marked by physical, cognitive, and psychosocial maturity, which affects how people feel, think, make decisions, and interact with their environment. As a result, it's a key time to establish the groundwork for their future investment's excellent health. This study believed that young people between the ages of 10 and 19 constitute an important population that requires access to health care that is affordable, accessible, acceptable, equitable, appropriate, and effective, as well as a safe and supportive environment, information about sexuality that is age-appropriate and comprehensive, and opportunities to develop life skills.

### Sample size and sampling procedure

**Fig. 1:**
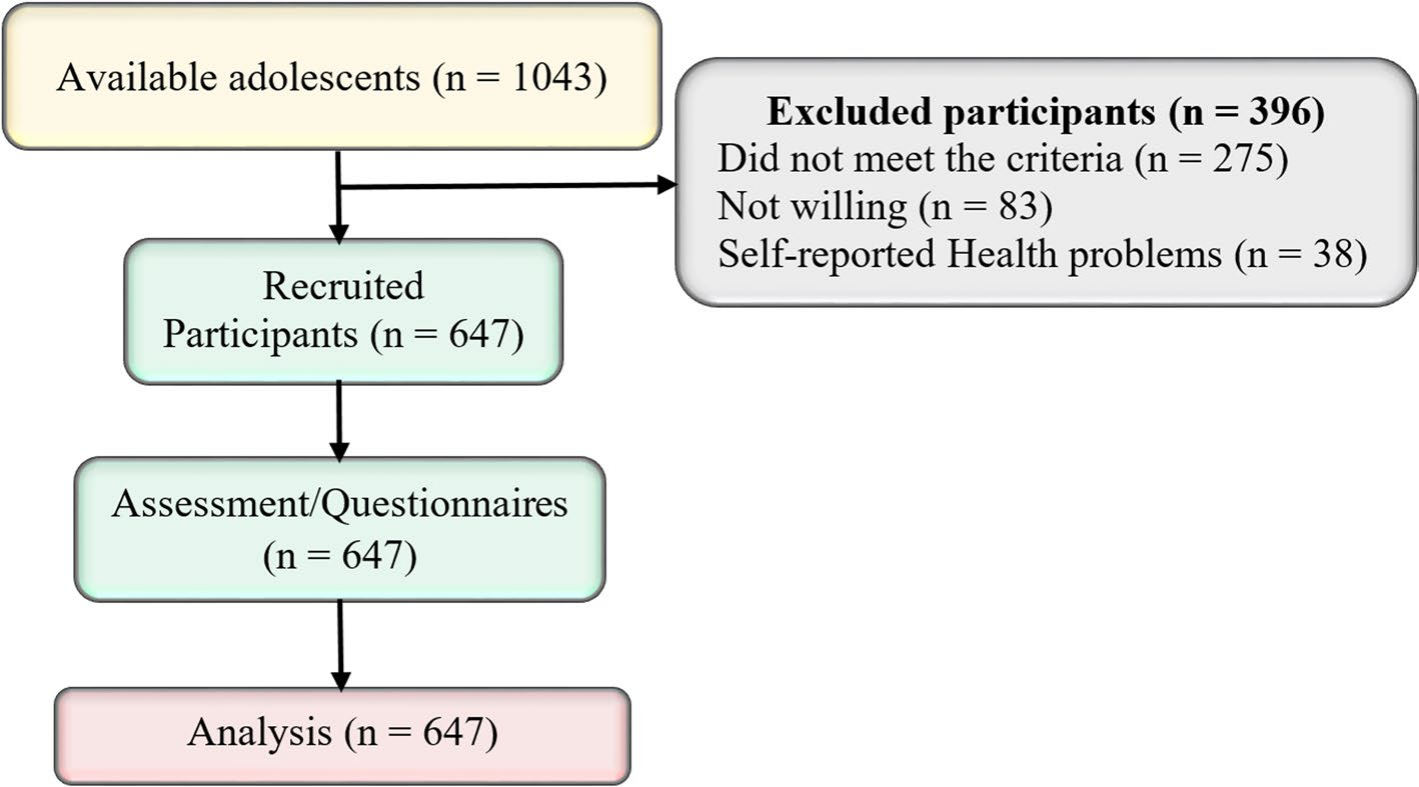
A sampling diagram showing the minimum sample size of the study. Source: Study plan (2020)

As used by other previous scholarly works [10, 38, 39], a simple random sampling technique by lottery method was employed to sample two of Tanzania's seven zones, including the central and coastal ones. The names of the zones were written on pieces of paper, which were then sealed and mixed in a box to perform a lottery sampling operation. Two items were chosen from the box for the study based on the established study minimum sample size and the accessibility of the study respondents. As shown in Fig. 1, sums of 647 (62.0%) out of 1043 adolescents consented and were recruited to participate in the study. However, 396 (38.0%) adolescents were excluded due to different reasons. Among them, 275 (69.4%) were excluded because they were form four adolescents in preparation for their final form four examination, 83 (21.0%) adolescents declined to join the study due to parental directives/consent, and 38 (9.6%) adolescents due to various self-reported health problems. A minimum sample size of 647 teens was produced using a random numbers table sampling technique, and classes were selected using a stratified random sampling procedure.

The sample was then evenly dispersed across the selected secondary schools in mainland Tanzania using the proportionate approach $(ni=Pi \times \frac{n}{P})$. Respondents in the study were only allowed to sign up voluntarily, so they were free to leave the study at any time without being questioned or having their information processed and evaluated. Respondents had to be accepted, registered, and either on-campus or off-campus during the semester at one of the sampled secondary schools. Adolescents living on the streets and those who had dropped out of school were excluded.

### Data collection instruments and procedures

The study collected primary information using 22 items of the pre-tested Sexual-Risk Behaviour Beliefs and Self-esteem Scale (SRBBSES) [38]. Tight, Mok, and Huisman [39] and Unis et al*.,* [40], recommended the adopted instrument when a researcher intends to assess sexual behaviours, beliefs, and self-esteem regarding safe sex among young people. The items had “Yes–No” responses of which “Yes” had a value of 1 score while the “No” response had a “0” score. For sexual behaviours “Yes” response represented the presence of any of the mentioned sexual behaviours in an item whereas “No” represented no sexual behaviours.

The sexual behaviours items were analytically converted to create a new variable called sexual ideology, which was then dichotomized into "Positive" and "Negative". Negative sexual ideologies are those ideas held by adolescents who believe that having relationships and engaging in sexual activity while they are adolescents is their right and has no negative health effects as long as people love each other. Other examples of those ideologies include the belief that risky sexual behaviour can result in teen pregnancies, new STIs/HIV infections, and school dropouts. The analytical transformation of the items that measured sexual behaviours produced a new end-point of analysing the variable that was dichotomized into "Yes" (demonstrated the behaviour) and "No." (Did not demonstrate any sexual behaviour). The school principal introduced the research assistant to the children before she was left in the classroom to offer the students greater autonomy and privacy to fill out the questionnaires within a separate unoccupied classroom. In one unoccupied venue of the sampled sample schools, 50 pupils may be seated at once with approximately one meter between chairs.

### Validity and reliability of the research tools

#### Validity

Content validity was opted and it was assured in this study in a way that questionnaires were anonymously filled out after being translated from English into the Swahili language to facilitate clarity and maximize understanding of the items among adolescents. Respondents’ names were not included in the questionnaires to ensure confidentiality. Referring to the approaches used by previous scholars [10, 30, 41–44] the tool was then shared with the supervisors, senior researchers, statisticians, and subject specialists for their technical and professional assistance on content validity, age appropriateness, and contextual appropriateness. All respondents’ responses in the questionnaires were secured confidentially in a keyed file by the principal investigator.

#### Reliability

The principal investigator conducted the pre-test of the research tools to a 10% (*n* = 65) sample of the calculated sample size in an independent geographical location from the sampled study settings and the determined sample size to prevent information contamination. Although there would be some biases, the pre-test's location was chosen based on geographical differences (Morogoro region) but to the sample that had almost similar characteristics to the study sample. Since the authors reasoned that keeping it not in the same area as the study would reduce physical contact and thus, communication about the content among adolescents during the actual field data collection. Indicators such as the relevance of the items, language appropriateness, clarity, and duration it would take to finish filling the questionnaires.

The inter-observer rating was employed to rate the relevance of the items among 10 consulted independent reviewers/observers. To ensure privacy, data-gathering processes were carried out in a separate, empty room on the respective school grounds with permission from the school heads. Observation from a pre-test revealed that all items were relevant with a score range of 9/10 to 10/10, the language was appropriate and clear and the questionnaires would be filled and completed within a range of 30 to 60 min. The Kaiser-Meyer-Oklin (KMO) value of ≥ 0.05 and p ≤ 0.05 was used to assess sample adequacy and it was set at a cut-off point of ≥ 0.60. The correlation coefficient was set at a cut-off point of ≥ 0.30. Additionally, the factorability of the correlation matrix was supported and the suitability of the original data for factor analysis was examined using Bartlett's test of sphericity. Findings of the pre-test were then subjected to a scale analysis to determine the reliability measure of the tools which revealed a Cronbach α = 0.77. Based on the recommendations by previous scholars [45–47] the tool was considered reliable for the actual field data collection.

### Data analysis

Statistical Analysis Software (SAS) computer program version 9.4 available in the institution was used for both descriptive and inferential statistical data analysis. A total of 647 (100% response rate) completed the study. Data were cleaned first and checked for normality as the criteria of opting for analytical measurements of which parametric measurements have opted for the approximately normally distributed data otherwise, non-parametric measurements. Socio-demographic characteristics profiles of the study respondents and the sexual ideology, patterns, prevalence, onset, and associated factors were analysed descriptively and presented in frequencies and percentages. The Chi-square test and cross-tabulation analysis established the relationship between variables, while the binary and multinomial logistic regression model was used to determine the association between predictor variables and the outcomes of interest under study which was set at a 95% confidence interval and 5% significance level.

The following logistic regression model was used

1 $$\lbrack p=\frac1{1+{e-}^{{(b}_0+b_1x)}}\rbrack(\leq0p\leq1)$$

Whereas; *Ƥ: the* predicted probability of an outcome.

*e:* Exponential.

*b*_*0:*_ Constant value.

*b*_*1*_*:* Slope.

*x:* predictor variable.

## Results

### Sociodemographic and other co-related characteristics profiles of the study respondents

The response rate of the study was 100%. Findings in Table 1 indicate that the mean age of the study respondents was 15 ± 1.869 years. The most prominent age group (71.2%) ranged from 13 to 16 years. Females constituted 57.5% (*n* = 372) of the sample. Very few (26.7%) respondents had opportunities to sometimes (17.3%) talk with their parents about SRH matters while 73.6% (*n* = 476) of them engaged in social groups, 98.6% (*n* = 638) of the study respondents are social media platforms. Study respondents who were abusing drugs accounted for only 12.8% (*n* = 83) of the studied sample. Other socio-demographic characteristics of the study respondents were found as shown in the table.

**Table 1:** Sociodemographic and other co-related characteristics profiles of the study respondents (*n* = 647)

Variable	n (%)
**Age in years**	
Mean Age in years = 15 ± 1.869	
Minimum in years = 12 years	
Maximum in years = 19 years	
Variable	
**Age Groups**	
10 to 12 yrs	58(9.0%)
13 to 16 yrs	461(71.3%)
17 to 19 yrs	128(19.8%)
**Sex**	
Male	275(42.5%)
Female	372(57.5%)
**Religion**	
Christian	195(30.1%)
Muslim	452(69.9%)
**Current year of study in school**	
First-year	275(42.5%)
Second-year	174(26.9%)
Third-year	198(30.6%)
**The education level of Father**	
Never gone to School	117(18.1%)
Primary Education	260(40.2%)
Secondary Education	174(26.9%)
College/University	96(14.8%)
**The education level of Mother**	
Never gone to School	163(25.2%)
Primary Education	310(47.9%)
Secondary Education	49(7.6%)
College/University	125(19.3%)
**Occupation of Father**	
Self Employed	541(83.6%)
Government/NGOs Employ	76(11.7%)
Not working	30(4.6%)
**Occupation of Mother**	
Self Employed	549(84.9%)
Government/NGOs Employ	39(6.0%)
Not working	59(9.1%)
**Parents living together in the same Household**	
Yes	361(55.8%)
No	286(44.2%)
**Type of family**	
Nuclear Family	337(52.1%)
Extended family	310(47.9%)
**Head of the family at Home**	
Father	500(77.3%)
Mother	73(11.3%)
Relative	74(11.4%)
**Other co-related characteristics**	
**Communicated with parents on SRH matters**	
Yes	173(26.7%)
No	474(73.3%)
**Financial Protection**	
Yes	211(32.6%)
No	436(67.4%)
**Status of Social Cohesion**	
Yes	476(73.6%)
No	171(26.4%)
**Exposure to Media**	
Yes	638(98.6%)
No	9(1.4%)
**Exposure to Drug Abuse**	
Yes	83(12.8%)
No	564(87.2%)

Source: Field data (2020)

### The attitude (Patterns of sexual ideology) towards sexual risk behaviours among the study respondents

**Fig. 2:**
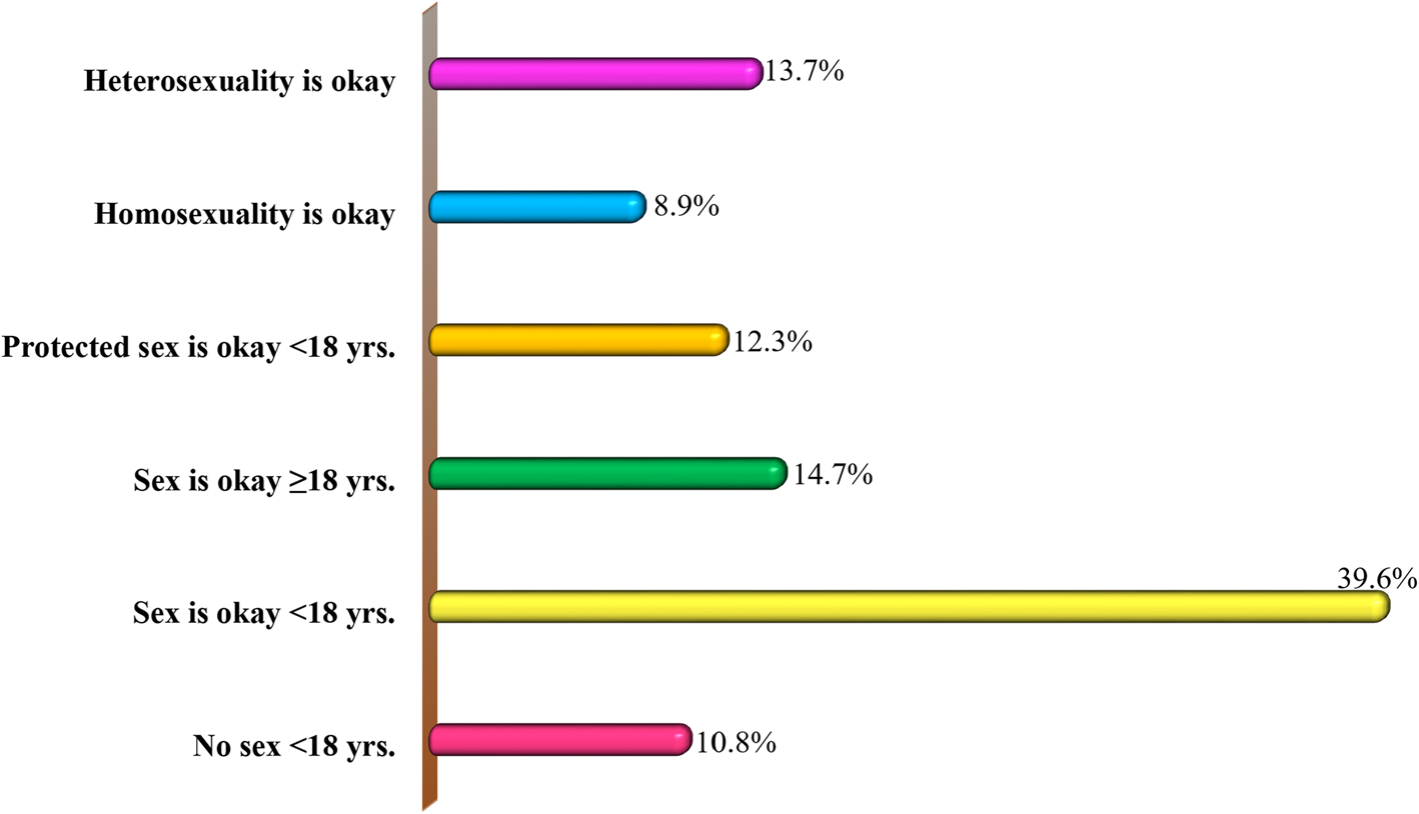
The attitude (Patterns of sexual ideology) towards sexual risk behaviours among the study respondents (*n* = 647), Key. Proportions ≥ 50%: Defined as significantly high proportions. Source: Field Data (2020)

Figure 2 shows descriptive findings of attitude (Sexual ideology) towards sexual risk behaviours among the study respondents of which 39.6% (*n* = 256) of them believed that sexual behaviours were okay even < 18 years of age against 14.7% (*n* = 95) study respondents who held the ideology that sexual behaviours are okay when they are ≥ 18 years of age. Adolescents below ( <) 18 years and equal to or greater than ( ≥) 18 years were age points used as the reference points to examine the study respondents’ sexual ideologies on the right time to initiate sexual activities. They were the options for the item “When is the right time for adolescents to start sexual activities?” Nevertheless, 13.7% (*n* = 89) of the study respondents reported that heterosexuality was much better against 8.9% (*n* = 58) of them who reported that homosexuality was okay to them. Study respondents who believed that protected sexual behaviours were okay in the adolescence stage < 18 years of age accounted for 12.3% (*n* = 80). However, some study respondents hold a belief that it is not appropriate for them to initiate sexual behaviours < 18 years.

### The prevalence and sexual debut (Onset of sexual risk behaviours) among the study respondents

**Fig. 3:**
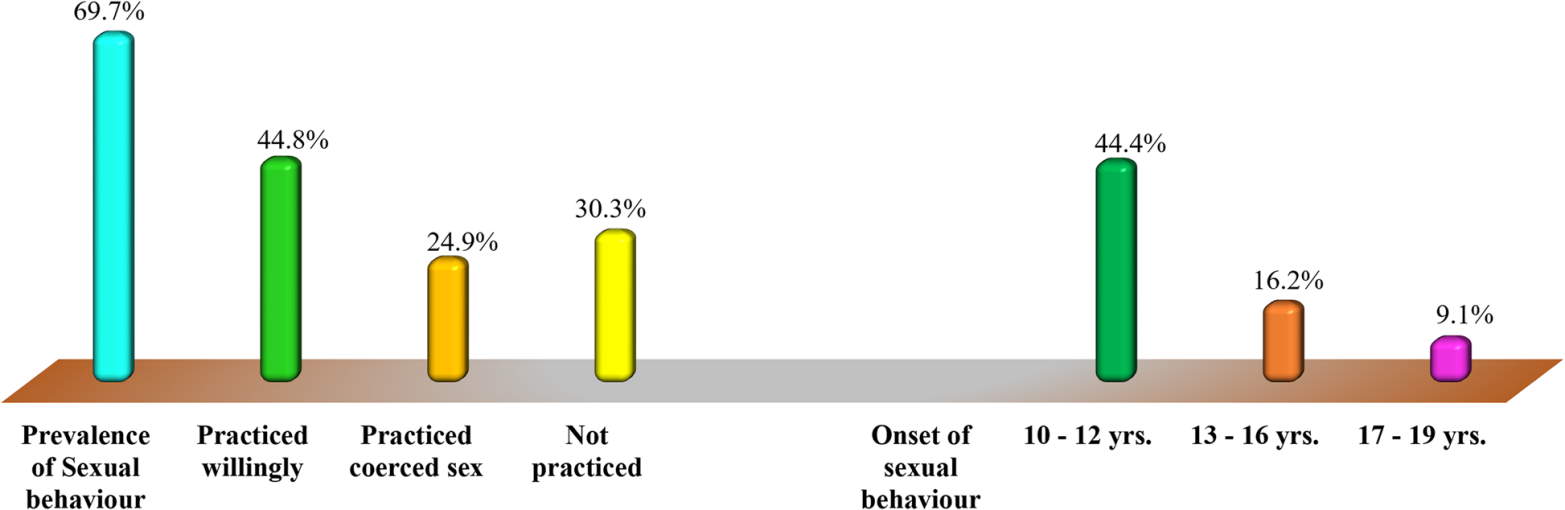
The prevalence and sexual debut (Onset of sexual risk behaviours) among the study respondents (*n* = 647). Key. Proportions ≥ 50%: Defined as significantly high proportions. Source: Field Data (2020)

As shown in Fig. 3, 69.7% (*n* = 451) of the study respondents involved themselves in sexual activities during their adolescence stage against 30.3% (*n* = 196) who did not. Study respondents who practised sexual behaviours based on their willingness to do so accounted for 44.8% (*n* = 202) while 24.9% (*n* = 112) of them involved in sexual activities by being coerced by either a friend or strange adults. Moreover, 44.4% (*n* = 200) of the study respondents who involved themselves in sexual behaviours initiated them between the age of 17 and 19 years, while 16.2% (*n* = 73) and 9.1% (*n* = 41) of them initiated sexual behaviours at the age between 13 and 16 years and 17 and 19 years respectively.

### Factors associated with the sexual risk behaviours among the study respondents

Multinomial logistic regression was performed to determine the association between variables. Table 2 indicates that with the control of other factors, the odds of being female on the onset of sexual behaviours were significantly high (AOR = 1.456; *p* < 0.05; 95%CI: 0.992, 3.906) against their counterparts males. The study findings yet revealed that early (10 to 12 years) and middle (13 to 16 years) adolescence stages were more times likely to initiate sexual behaviours by (AOR = 1.227; *p* < 0.05; 95%CI: 0.984, 4.321) and (AOR = 1.207; *p* < 0.05; 95%CI: 0.844, 4.003) respectively. Study respondents who were living in extended families (AOR = 1.009; *p* < 0.05; 95%CI: 0.503, 2.052); families headed by relatives (AOR = 1.002; *p* < 0.05; 95%CI: 0.178, 2.887); living with parents/relatives who were not working/having waged works (AOR = 1.403; *p* < 0.05; 95%CI: 0.965, 3.353); being not financially protected by parents/relatives (AOR = 1.007; *p* < 0.05; 95%CI: 0.983, 2.652) and respondents who communicated sexual and reproductive health matters with their parents/relatives (AOR = 1.782; *p* < 0.05; 95%CI: 0.879, 3.111) were significantly at high risk of practicing sexual behaviours at their adolescence stage than others. Nevertheless, study respondents who engaged in social groups (AOR = 1.032; *p* < 0.05; 95%CI: 0.889, 2.044); were exposed and using social media (AOR = 1.006; *p* < 0.05; 95%CI: 0.748, 2.667) and the exposure and use of drug abuse (AOR = 1.210; *p* < 0.05; 95%CI: 0.803, 2.130) demonstrated significantly high odds over adolescents’ sexual behaviours as compared with others.

**Table 2:** Factors associated with the sexual risk behaviours among the study respondents (*n* = 647)

Variable	COR	*p*-value	95%CI		AOR	*p*-value	95%CI	
			**Low**	**Up**			**Low**	**Up**
**Age Groups**								
10 to 12 yrs	2.045	0.003	1.031	5.112	1.227	**0.041**	0.981	4.321
13 to 16 yrs	2.975	0.033	1.062	5.771	1.207	**0.032**	0.844	4.003
17 to 19 yrs	1							
**Sex**								
Male	1							
Female	2.438	0.008	1.062	4.793	1.456	**0.027**	0.992	3.906
**Current year of study in school**								
First-year	3.645	0.001	1.887	6.067	2.056	**0.011**	1.021	5.329
Second-year	2.902	0.031	1.117	5.032	1.440	**0.043**	0.903	4.103
Third-year	1							
**The education level of Father**								
Never gone to School	2.667	0.021	1.024	4.472	1.128	0.146	0.398	3.337
Primary	1.984	0.059	0.897	2.671	1.043	0.063	0.652	1.873
Secondary	0.897	0.061	0.212	1.897	0.789	0.102	0.107	1.669
College/University	1							
**The education level of Mother**								
Never gone to School	2.673	0.012	1.249	5.320	1.874	0.086	0.365	3.210
Primary	1.478	0.042	0.983	3.764	0.891	0.078	0.452	2.045
Secondary	0.897	0.059	0.304	1.889	0.612	0.063	0.065	1.873
College/University	1							
**Occupation of Father**								
Self Employed	1							
Government	1.037	0.062	0.979	3.320	0.874	0.136	0.365	2.210
NGOs	1.076	0.072	0.731	2.764	0.819	0.108	0.352	1.784
Not working	1.897	0.091	0.607	2.908	0.903	0.068	0.265	2.873
**Occupation of Mother**								
Self Employed	1							
Government	0.799	0.062	0.979	3.320	0.874	0.136	0.365	2.210
NGOs	1.671	0.102	0.800	3.478	0.910	0.113	0.322	2.411
Not working	2.897	0.021	1.107	4.908	1.403	**0.038**	0.965	3.353
**Parents living together in the same Household**								
Yes	1							
No	2.738	0.013	1.087	4.002	1.324	**0.037**	0.593	2.035
**Type of family**								
Nuclear	1							
Extended	1.784	0.032	0.992	3.221	1.009	**0.041**	0.503	2.052
**Head of the family at Home**								
Father	1							
Mother	0.758	0.067	0.305	2.012	0.691	0.082	0.173	1.789
Relative	1.771	0.037	0.874	2.589	1.002	**0.041**	0.178	2.887
**Communicated with parents on SRH matters**								
Yes	1							
No	2.806	0.013	1.056	5.221	1.782	**0.034**	0.879	3.111
**Financial Protection**								
Yes	1							
No	1.973	0.026	0.987	3.477	1.007	**0.040**	0.983	2.652
**Status of Social Cohesion**								
Yes	1.873	0.038	0.794	3.214	1.032	**0.045**	0.889	2.044
No	1							
**Exposure to Media**								
Yes	1.769	0.039	0.778	3.031	1.006	**0.049**	0.748	2.667
No	1							
**Exposure to Drug Abuse**								
Yes	2.007	0.011	0.892	4.712	1.210	**0.033**	0.803	2.130
No	1							
**Attitude (Sexual ideologies)**								
Heterosexuality	1.939	0.028	0.793	3.113	1.020	**0.042**	0.732	2.307
Homosexuality	0.983	0.065	0.343	1.998	0.830	0.071	0.313	1.898
Protected sex < 18 yrs	2.305	0.011	1.721	4.559	1.435	**0.037**	0.881	2.769
Sex is okay ≥ 18 yrs	2.093	0.032	1.036	4.334	1.103	**0.043**	0.879	2.678
Sex is okay < 18 yrs	2.421	0.035	1.528	4.212	1.293	**0.048**	0.689	2.989
No sex at < 18 yrs	1							

Source: Field data (2020)

COR ≥ 1 and *p* < 0.05: a positive predictor of the outcome variable when not controlled with other factors

AOR ≥ 1 and *p* < 0.05: a positive predictor of the outcome variable when controlled with other factors (The final finding to be reported)

*p* < 0.05: Significant association between variables

## Discussion

The results of this study showed that while some adolescents had the belief that they should abstain from sexual activity until they reached adulthood, the majority of them had unfavorable sexual ideologies regarding the beginning of sexual activity during the adolescent phases. Some of them thought it was alright for them to participate in sexual behaviour even though they were still too young to do so, while others thought it would be preferable if they slept with someone of the opposite sex. However, several teenagers claimed that they felt comfortable engaging in sexual activity with a buddy who is also of the same sex. Linked with other studies’ findings [1, 10], the findings of this study may suggest that the majority of the study's respondents were not adequately informed about age-appropriate, comprehensive sexual and reproductive health issues, as well as health services for delayed initiation and/or safe sexual behaviours during adolescent stages. Similarities in findings between studies may be attributed to the matching in the topic under study, respondents’ demographic factors, and methodology.

In this study, there was a strong correlation between teenage sexual ideology and the prevalence of sexual behaviour among adolescents. The majority of adolescents engaged in sexual activities voluntarily as opposed to the handful who claimed to have done so under duress from classmates or unknown adults. The results might suggest that the adolescent stage is malleable in terms of analysing, synthesizing, identifying, and making informed and responsible decisions about sexual behaviours. As a result, they could succumb readily to pressure from peers or adults to engage in sexual activity, whether they want to or not. Needless to say, as observed by some previous scholarly works [5, 48, 49], the results of this study have shown that the early onset of sexual behaviours among adolescents (whether it be sexual relationships with peers and adults or having sexual encounters with one or more partners) is prominent enough to be recognized as a problem of public concern that may need to be appropriately addressed to promote sexual health and safety to young people. Issues such as research variables, respondents, theory, and technique may have contributed to the similar findings observed in this study to those from the previously cited studies.

The majority of adolescents in this study began engaging in sexual behaviours between the ages of 10 and 17, which may indicate that the early and middle stages of adolescence were probably more strongly influenced by sexual emotions and motivated by the sexual ideologies that it was acceptable to engage in heterosexual or homosexual behaviours and/or it was adolescents’ rights having protected sex before the age of 18 years. This study makes a similar case as it has been made by some other previous scholarly works [50, 51] that early initiation of sexual behaviours in adolescents may be related to misinformation about comprehensive sexual and reproductive health information, education, and related health services from age-appropriate, controlled, guided, and reliable sources. The circumstance may suggest that young people need a continuum of parental guidance, support, and mentoring from close relatives, friends, teachers, religious leaders, and/or significant others to advocate for their rights to sexual and reproductive health for preventing or delaying the onset of risky sexual behaviours.

The study's findings also showed that adolescents who were exposed to drugs, alcohol, and social groups and who talked to their parents or other family members about sexual and reproductive issues were more likely than others to engage in sexual behaviours. This finding is discussed in the current study because the aforementioned elements would expose adolescents to unrestricted and improper sexual information and education, which would encourage them to copy and/or try the behaviours behind the backs of their caregivers. The study at hand ties the aforementioned factors to the incidence and onset of sexual behaviours because, in the absence of adequate parental supervision and ongoing support, adolescents would be more likely to succumb to their sexual urges and lose self-control. This would be the case because, according to other scholars, a person's sociodemographic characteristics profiles and sexual ideologies toward oneself are thought to be significantly associated with engaging in unjustified and irresponsible sexual behaviours and practicing self-rejection [33].

As discussed by previous scholars [48, 49], Adolescent sexual and reproductive health is closely monitored, controlled, supported, and communicated with by parents when there is a positive parent-adolescent connection. Teenagers in this study who expressed a negative sexual ideology likely lacked the parental support necessary to be financially secure. This conclusion was related to the sociodemographic backgrounds of the teenagers, which revealed that about 44.2% of the studied adolescents did not live in the same household as their parents. This suggests that the more financially secure a child's parents are, the more accepting of risky sexual behaviour they become since they are less likely to be taken advantage of or enticed to engage in the behaviour for selfish reasons [50]. Nalukwago et al*.* [51]*,* in Uganda, discovered that the majority of teenagers who engaged in risky sexual behaviours had parents who spent the majority of their time at work.

According to the current study's discussion, the issue was deemed to have been caused by the parents' inadequate kid protection. In addition, adolescents in their second and third years of study were better equipped to form a positive sexual ideology than those in their first year. Under Schiller's descriptions [52] on human neuro-maturation, a person's perspective toward events might change depending on how much exposure s/he receives to them. Additionally, Wilson et al*.* [53]*,* early adolescents showed lower levels of resilience than their older counterparts, according to a cross-sectional and explanatory study about resilience on sexual risk behaviours of STI among adolescents. Once burned by fire, a growing child can quit playing with it. The experience of the fire's unfavorable health effects, which led to the development of a negative attitude toward it, causes the person to cease [54–56]. The second and third-year teenagers would have encountered and/or grown accustomed to dangerous sexual behaviours at some point in their life routes, and they would have gradually evolved a positive attitude about it as being detrimental to their sexual health [57]. Thus, the findings of this study and other scholarly works agree that investing in adolescents for age-appropriate comprehensive sexual and reproductive health information, education, and associated health services such as family planning strategies and career capacity building for their job opportunities appears to be very important and timely.

## Conclusion

Findings suggest that holding a positive attitude towards early sexual debut is a precursor to early sexual activity among adolescents. Unsafe sex, coercive sex, and other risky sexual behaviors are not uncommon among adolescents starting sex before the age of 18 years. Exposure to drug abuse, online sexual content, and/or social groups significantly influenced early sexual debut irrespective of other known factors. The majority of teenagers, as indicated by the aforementioned findings, held a negative sexual ideology and felt that it was acceptable for them to engage in sexual activity while still in adolescence. Their ideologies were directly related to the beginning of sexual behaviour before the age of 18, and the majority of them began sexual behaviour earlier and in the middle of adolescence rather than later on. Even if they engaged in sexual behaviours of their own free will, some were coerced into doing so by adults or other peers. Their sexual philosophies and sociodemographic profiles led them to engage in sexual activity without making an informed, responsible decision before the act with either peers or adults who were unfamiliar to them. The results imply that inappropriate sexual behaviours among adolescents are a complex, pervasive behavioural issue that requires in-depth research that takes into account both an individual's internal and external circumstances.

Age-appropriate school-based sexuality education programs should be promoted and implemented to address the most prevalent positive attitude towards early sexual debut and associated risk sexual behaviour among adolescents in Tanzania and other similar settings. When developing an innovative sexual-based educational intervention, projects, and or programs that focus on involving a cohort of young people as the key players in addressing sexual and reproductive health issues around them, it is important to analyse the parental guidance and support, educational environment, and other factors adolescents are exposed to. The results of this study are pertinent to the creation of prevention programs and/or treatments for sexual and reproductive health that may improve the communication of sexual and reproductive health information to adolescents. The results of this study offer opportunities for education and health professionals to contribute and focus efforts to help enhance the current curricula and/or syllabus in a way that supports and shapes adolescents' sexual ideologies for safe sex. This study makes recommendations for more studies to plan, create, and carry out safer sexual interventions with young people. Teenagers may get the ability to make educated and logical decisions about sexual activity if this is done.

## Strength of the study

The findings of this study have answered the research gap presented in the introduction Sect. (1,4,30]. Studying sexual ideology among adolescents may add new knowledge to the existing comprehensive sexual education and youth programs already in place. This study addressed an issue of public concern, including early onset of sexual activities among adolescents, unsafe sexual behaviours that expose them to teenage pregnancies and/or Sexually Transmitted Infections (STIs), and incidences of Human Immunodeficiency Virus (HIV). The results of this study may serve as the basis for further interventional research or significant initiatives aimed at addressing sexual and reproductive health problems in teenagers.

## Implications for Practices and Future Research

Similar to what other previous studies [2, 4] have highlighted, the findings of this study can be used to develop unique strategies for involving and empowering adolescents with good sexual ideas against sexual activities. If the findings of this study are published in several scholarly journals, they will provide a useful understanding of the topic under study for further innovative and action research.

## Limitations of the study

Similarly to what has been reported by previous scholarly works [1, 33, 41], the study findings may not be generalised to other geographical locations of Tanzania. Moreover, the study findings represent in-school adolescents only thus, they may not be represented and interpreted for out-of-school adolescents too because of the ethical issues especially the collection of written informed consent and difficulties in gathering and handling them in one point for the study. It is also probable that dependability, transferability, and/or confirmability rigor were not taken into account in this analysis because the study did not use a triangulation approach for data collection by taking into account ethical clearance and permit that was offered by the Institutional Research Review Ethics Committee and the Ministry of education. Moreover, adolescents would have had trouble recalling and sharing their prior lived experiences about sexual behaviours, so the study's findings may need to be interpreted cautiously. The ability to rate oneself presents a dilemma since it could cause someone to underestimate, exaggerate, or report the actions or data of the study's respondents. Therefore, caution should be used in interpreting the findings of this study.

## Data Availability

Available under request at walter.millanzi@udom.ac.tz because further analyses of other variables are being performed.
